# Nondisclosure of Financial Interest in Clinical Practice Guideline Development: An Intractable Problem?

**DOI:** 10.1371/journal.pmed.1002030

**Published:** 2016-05-31

**Authors:** Hilda Bastian

**Affiliations:** National Center for Biotechnology Information, National Library of Medicine, National Institutes of Health, Bethesda, Maryland, United States of America

## Abstract

In a Perspective linked to Stelfox and colleagues, Hilda Bastian discusses the challenges of improving transparency and management of financial conflicts of interest among committees that develop guidelines for medical practice.

This week in *PLOS Medicine*, Stelfox and colleagues [1] report that only half of a large sample of clinical practice guidelines included financial disclosure statements for committee members (51%). They were studying 290 national or international practice guidelines for medical professionals published by 95 organizations in the United States National Guideline Clearinghouse in 2012.

That’s about the same rate that Taylor and Giles found in 2004 [2] and Norris and colleagues found in 2010 [3]. There may be even less transparency across the whole spectrum of clinical practice guidelines. The National Guideline Clearinghouse includes a selected group, all published in English. It’s hard to know how reflective those are of guidelines generally. In the late 1990s, when Grilli and colleagues studied guidelines identified via MEDLINE, they found that 67% did not report any description at all of the professionals involved in developing the guidelines [4]. A 2013 study of Danish specialty societies found that only one out of 45 guidelines disclosed financial interests the authors identified from an official national disclosure list [5], and a 2015 study of primary care guidelines found no statements about conflicts of interest in 69% [6].

When guideline recommendations are controversial—and that’s often—suspicion quickly turns to financial conflicts of interest. The perception of conflicts can call the reliability of a recommendation into question, and even more so if there was no disclosure. This new study adds fuel to those concerns. Stelfox and colleagues found that organizations with weaker policies on financial conflicts tended to make more positive recommendations about the use of biomedical products.

## Policy on Management of Conflicts of Interest Is Getting Stronger, But Is It Enough?

Clinical practice guidelines need to be based on solid scientific grounds and expertise. However, the science, the experts, and the organizations developing guidelines can have major financial entanglements—and that can be true of the best experts and research in the area. Managing potential conflicts well is tough in this context, but it’s one of the most essential steps to making a guideline both credible and trusted.

With hindsight, I think those of us encouraging better methodology for guideline development in the 1990s took the issue of disclosure of financial interests too much for granted. It seemed so self-evident, it got barely a mention even in national policy on guideline development [7]. Policies have been getting more detailed and much stronger, however.

The US Institute of Medicine took a strong position on this the year before the guidelines evaluated in Stelfox and colleagues’ study were published. In general, the Institute of Medicine report concluded, the quality of guidelines’ “development processes and guideline developer adherence to quality standards have remained unsatisfactory and unreliable for decades” [8]. Last year, the Guidelines International Network took a strong position on the need to improve management of conflicts of interest as well [9].

It’s hard to know, though, whether we should feel confident that adherence to these policies on conflicts of interest will be better than adherence to other quality standards have been—like those on the evaluation of research [8]. That is a particular concern with increasing pressure to speed up the guideline development process and to reduce its costs [8].

## Less Visible Organizational and Personal Financial Interests

Stelfox and colleagues focus particularly on the organizational conflicts of interest of guideline producers and their policies. They examine the financial interests of the organizations, but not of the individuals employed within those organizations. This same blind spot is evident when it comes to policies about committee members; the financial interests of the organizations that individuals represent tend to be disregarded. Yet these can be substantial, including for patients’ organizations.

Of organizations responding to the Stelfox survey questions about managing conflicts of interest, most had policies, but their compliance with them often fell short. For example, of those reporting that a majority of guideline committee members must be free of financial conflicts, Stelfox and colleagues found that 61% produced at least one guideline in which a majority of the members disclosed company relationships.

These less visible lines of potential influence could be having more of an impact than we realize. The influence of staff members, of those who assess and manage the data presented to committee members, and the role of the chairperson could be pivotal. Graham and colleagues undertook a qualitative study of the management of conflicts of interest at the UK National Institute for Health and Care Excellence [10]. Their work pointed to the level of invisibility and unawareness of the potential for conflict among those participating in guideline development. Policy is not enough, according to Graham and colleagues. Successful implementation will require more clarity in policy and procedures, as well as training of chairpersons and evaluation of practice.

Moynihan and colleagues opened up another line that has not had the visibility of evaluation of, and recommendations about, biomedical products: expanding the definitions of disease [11]. They studied 16 guidelines by widely recognized US-based organizations on common conditions, identified from a search of MEDLINE, the National Guidelines Clearinghouse, and the National Institutes of Health website. Of those, ten proposed a widening of disease definition, by formulating pre-disease conditions or lowering a diagnostic threshold, for example. There were financial disclosures for all but two of the guidelines, and the average proportion of members with industry connections was 75%, including the chairs of 12 development committees.

Studies are expanding and deepening our understanding of the influences on clinical practice guidelines of interests that run counter to those of patients. This research will no doubt be helpful to the organizations who are already taking conflict of interest management seriously. They will keep improving. But those organizations are not why this problem seems to be intractable.

Guideline processes without adequate financial conflict management have to become unacceptable to a far wider circle. They need to become unacceptable to influential committee members, to the medical journals that lend so many guidelines additional standing and reach, and to the membership of the professional societies that produce them. Until that happens, for guidelines as for clinical research, it’s a case of *caveat lector*: let the reader beware [12].

## References

[1] Stelfox HT, Campsall P, Colizza K, Straus S. A cross-sectional study of financial relationships between organizations that produce clinical practice guidelines and the biomedical industry. PLOS Med. 2016;13:e1002029 10.1371/journal.pmed.1002029 27244653 PMC4887051

[2] Taylor R, Giles J. Cash interests taint drug advice. Nature. 2005;437(7062):1070–1071. 16237402 10.1038/4371070a

[3] Norris SL, Holmer HK, Ogden LA, Selph SS, Fu R. Conflict of interest disclosures for clinical practice guidelines in the National Guideline Clearinghouse. PLOS ONE. 2012;7(11):e47343 10.1371/journal.pone.0047343 23144816 PMC3492392

[4] Grilli R, Magrini N, Penna A, Mura G, Liberati A. Practice guidelines developed by specialty societies: the need for a critical appraisal. Lancet. 2000;355(9198):103–106. 10675167 10.1016/S0140-6736(99)02171-6

[5] Bindslev JBB, Schroll J, Gøtzsche PC, Lundh A. Underreporting of conflicts of interest in clinical practice guidelines: cross sectional study. BMC Med Ethics. 2013;14:19 10.1186/1472-6939-14-19 23642105 PMC3651727

[6] Allan GM, Kraut R, Crawshay A, Korownyk C, Vandermeer B, Kolber MR. Contributors to primary care guidelines: what are their professions and how many of them have conflicts of interest? Can Fam Physician. 2015;61:52–58. 25609522 PMC4301766

[7] National Health and Medical Research Council. A guide to the development, implementation and evaluation of clinical practice guidelines Canberra: Commonwealth of Australia; 1999 https://www.health.qld.gov.au/cpcre/pdf/nhmrc_clinprgde.pdf

[8] Institute of Medicine (US) Committee on Standards for Developing Trustworthy Clinical Practice Guidelines. Clinical practice guidelines we can trust. Washington (DC): National Academies Press; 2011.

[9] Schünemann HJ, Al-Ansary LA, Forland F, Kersten S, Komulainen J, Kopp IB, et al, for the Board of Trustees of the Guidelines International Network. Guidelines International Network: principles for disclosure of interests and management of conflicts in guidelines. Ann Intern Med. 2015;163:548–553. 10.7326/M14-1885 26436619

[10] Graham T, Alderson P, Stokes T. Managing conflicts of interest in the UK National Institute for Health and Care Excellence (NICE) clinical practice guidelines programme: qualitative study. PLOS ONE. 2015;10(3):e0122313 10.1371/journal.pone.0122313 25811754 PMC4374927

[11] Moynihan RN, Cooke GPE, Doust JA, Bero L, Hill S, Glasziou PP. Expanding disease definitions in guidelines and expert panel ties to industry: a cross-sectional study of common conditions in the United States. PLOS Med. 2013;10(8):e1001500 10.1371/journal.pmed.1001500 23966841 PMC3742441

[12] Bastian H. ‘They would say that, wouldn’t they?’ A reader’s guide to author and sponsor bias in clinical research. J R Soc Med. 2006;99(12):611–614. 17139062 10.1258/jrsm.99.12.611 PMC1676333

